# Study on the Characteristics of Circumferential and Longitudinal Flow of Vault Concrete during Tunnel Lining Pouring Processes

**DOI:** 10.3390/ma17030678

**Published:** 2024-01-31

**Authors:** Shuai Yang, Yimin Wu, Zhuangzhuang Zhou

**Affiliations:** 1 School of Civil Engineering, Central South University, Changsha 410075, China; 224811051@csu.edu.cn; 2 China State Construction International Investments Limited, Nanjing 211100, China; zzz15162122893@163.com

**Keywords:** tunnel engineering, pouring of the arch lining, fresh concrete, flow characteristics

## Abstract

With a large number of railroad and highway tunnels opening for operation, the diseases caused by hidden lining defects are increasing. The study of flow characteristics of freshly mixed concrete during tunnel lining casting is the key to revealing the formation mechanism of hidden defects. This paper revealed the location of blank lining formation by investigating the circumferential and longitudinal flow characteristics of concrete in the vault during tunnel pouring to provide suggestions for improving the quality of tunnel lining pouring for the various projects. This paper adopted the method of indoor testing, selected the suitable working conditions and flow parameters, validated the accuracy of the test with a numerical simulation, and simulated the secondary lining pouring process of the tunnel arch from the circumferential direction and longitudinal direction. This revealed the flow characteristics of the freshly mixed concrete in the process of pouring the arch lining. The flow of concrete in the arch lining was basically characterized by two major features which were similar to the flow in the pumping pipe and the layered flow. It also revealed the relationship between the concrete flow rate, flow distance, and the location of the formation of the blank lining risk zone with the slump of the concrete, the pumping pressure, and the radius of the tunnel.

## 1. Introduction

As the most familiar material in civil engineering, concrete is widely used in the construction of various infrastructure projects, especially tunnels. However, due to the fact that the main structure is located underground, the structural stress is complex, the quality assurance problem is a huge challenge, and the quality of the tunnel lining is particularly important [1,2]. According to the research statistics in recent years, about 10–30% of the tunnels built according to the existing process conditions have the phenomenon of void lining in the inspection process during operation [3], and some tunnels are accompanied by various forms of cracks [4]. These factors have caused certain impacts on the durability and strength of the structure, and produce serious hazards to the tunnel traffic.

For the various quality problems exposed by the secondary lining of mountain tunnels, many scholars have summarized a large number of experiences from the concrete lining [5] such as the compression damage of concrete lining [6], concrete lining maintenance temperature [7], concrete lining stress [8], concrete lining thickness insufficiency [9], the preparation of high-performance concrete [10], and the concrete lining pressure grouting technology [11] so as to reach a conclusion regarding the prevention and management system for dehiscence, cracks, and other diseases. Although the current management of engineering quality problems has achieved remarkable results [12], to solve the quality problems of mountain tunnel casting from the root, it is necessary to start from the flow characteristics of concrete itself in order to reveal the flow characteristics of freshly mixed concrete during the lining casting process and then grasp the root of the problems.

With the continuous improvement of computer numerical simulation technology, research methods relying on numerical simulation to analyze concrete flow properties have gradually emerged. From the point of view of the calculation method of the structure, the current simulation of concrete flowability is mainly based on two categories: finite element [13,14,15] and discrete element [13,16]. From the point of view of the simulation method, the simulation based on computational fluid dynamics (CFD) is one of the more dominant methods at present [17]. In recent years, emerging computational methods such as LBM [18] and SPH [19] have also been gradually applied to the process of numerical simulation of concrete flow, however these two simulation methods are not mature enough as of right now, and are still in the development stage.

Currently, there are many numerical simulation methods for concrete flow and the simulated engineering application scenarios are relatively wide, but the simulations and analyses of concrete flow are not deep enough in terms of individual projects. Especially in the construction process of mountain tunnels, the quality of lining casting is key for the safe operation of the subsequent tunnels, and there are fewer simulations of the concrete flow state during the tunnel lining casting process. This results in a lack of sufficient understanding of the flow characteristics of the concrete in the lining formwork, and has brought about an obstacle to the quality assurance of lining casting. Therefore, the use of numerical simulation methods to systematically reveal the flow characteristics of concrete in the lining formwork are an important guarantee to improve the quality of lining pouring. After comprehensive consideration, this paper used ANSYS FLUENT 2022R1 software (ANSYS, Pittsburgh, PA, USA) and CFD Eulerian multiphase flow simulation technology to carry out the relevant numerical simulation work.

## 2. Selection and Accuracy Verification of Rheological Parameters

In this section, rheometer tests were carried out with the aim of providing a reference for the selection of rheological parameters for subsequent numerical simulations. Slump extensibility tests and L-box flow tests were carried out in order to verify the accuracy of the rheological parameters obtained from the tests in the form of numerical simulations.

### 2.1. Concrete Flow Test

#### 2.1.1. Test Raw Materials

1 Concrete

Ordinary silicate cement with the mark of P.O42.5 produced by a factory in Hunan Province, China was used in this test. Its main performance indexes are shown in Table 1.

2 Fine aggregate

Mechanical medium sand from Zone II with a chloride ion content of less than 0.05% was selected as the fine aggregate for the test. The main performance indexes of medium sand are shown in Table 2 below.

3 Coarse aggregate

The test was conducted using coarse aggregate with a grain size range of 5–25 mm, a crushing value of 7.0%, and a needle flake content of 2.1%, with a total of 5–10 mm continuous graded small stones and 10–25 mm continuous graded medium stones.

4 Fly ash

In this paper, the test was carried out using Class II fly ash produced by a factory in Hunan Province, China. Its main performance indexes are shown in Table 3, in which all the indexes of Class II fly ash meet the specification requirements.

5 Water reducing agent

The test material was selected as a high-performance water reducing agent of polycarboxylate with a water reduction rate of up to 40%, PH value of 7–9, and white powder. The manufacturer is the Jinnite Environmental Protection Technology Limited Liability Company of Shandong Province, China.

6 Water

The water used for the test materials was tap water from the Hunan Province area in China.

#### 2.1.2. Test Working Conditions

A total of eight working conditions were selected for this test. M1 was the concrete proportioning scheme for the actual tunnel secondary lining poured on site, M2, M3, M1, M4, and M5 were different in that the water–cement ratio gradually increases, while the other parts were the same in order to analyze the effect of the water–cement ratio on the flow test. M6, M7, M1, and M8 were different in that the sand rate gradually increases, while the other parts were the same in order to analyze the effect of the sand rate on the flow test, as follows Table 4:

#### 2.1.3. Brief Description of Tests and Results

1 Rheometer test

**Figure 1:**
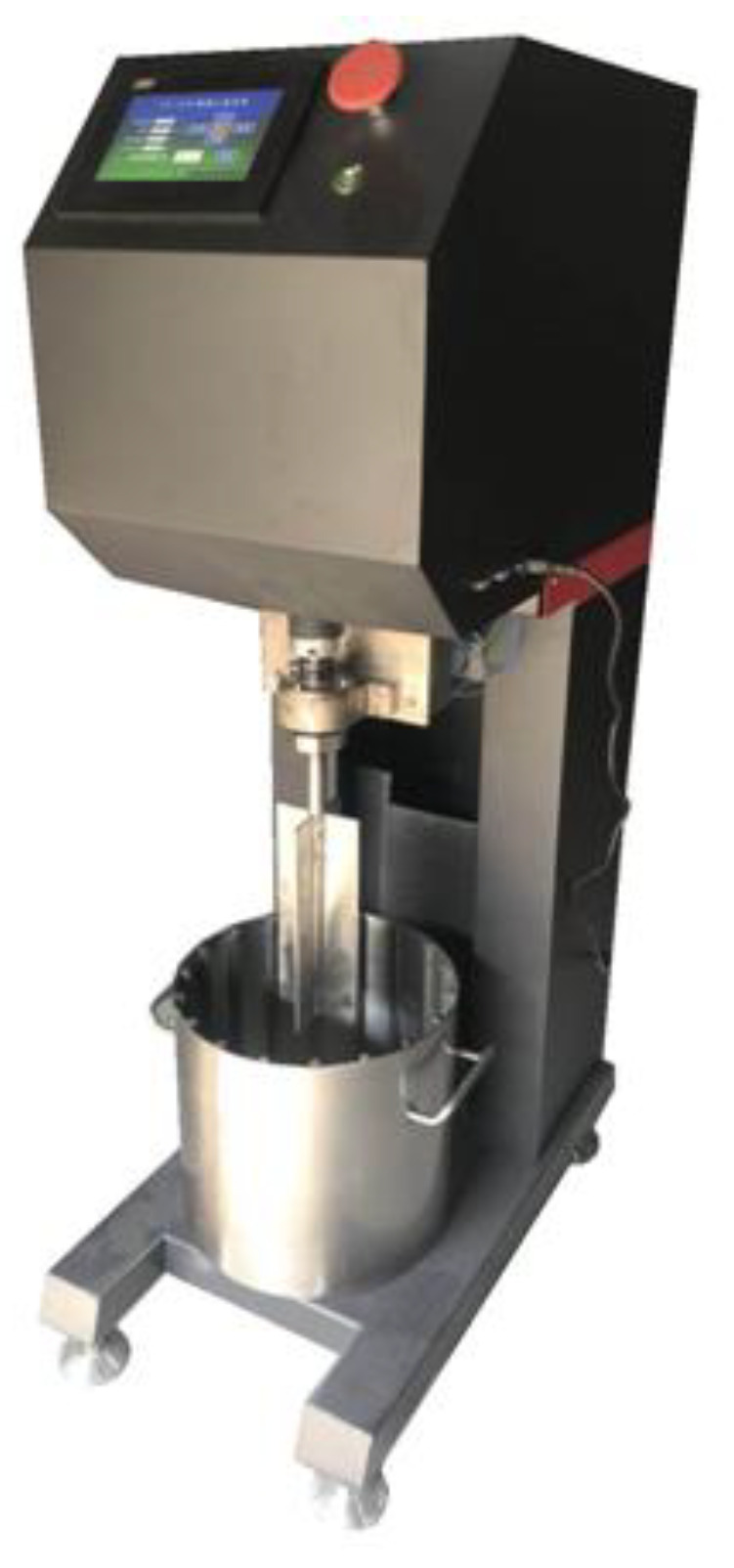
TR-CRI rotational rheometer.

Through comprehensive comparison, the TR-CRI concrete rotational rheometer produced by a manufacturer in Shanghai, China was selected for this paper, as follows Figure 1:

The diameter of the cross type rotor can be customized as required, and the standard size (diameter of 100 mm) was chosen for this test program. The experimental procedure is not described in detail here, and the rheological parameters of each group of specimens were obtained through calculation and fitting as shown in the Table 5:

2 Slump extension test

The slump cylinder used in this article is an iron round table cylinder with overall dimensions of 100 mm × 200 mm × 300 mm (top bottom × bottom bottom × height). The thickness of the cylinder wall is 1.5 mm, the inner side of the cylinder is smooth, and the slump cylinder is equipped with two handrails on the top and two pedals welded on the bottom, preventing the device from deviating from the center or detaching from the bottom plate in the process of the test. The slump extensometer is a steel plate of 1000 mm × 1000 mm with different diameter sizes engraved on the surface and a thickness of 2.0 mm.

Following the standard slump and extensibility test procedure, three valid values were taken for each condition and averaged. The test results are obtained as shown in Table 6, with no further details of the process.

3 L-box flow test

**Figure 2:**
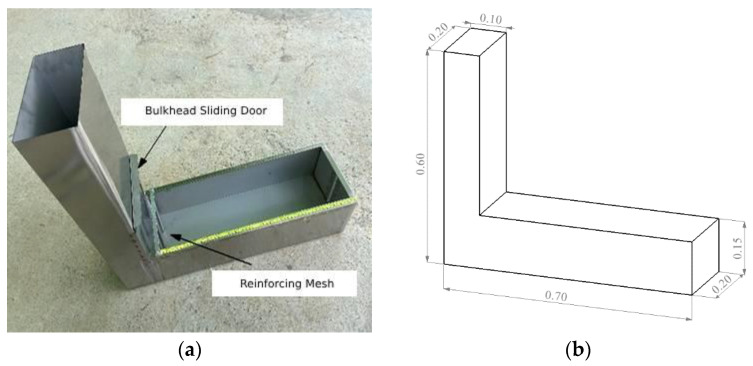
(**a**) Physical drawing and (**b**) L-box dimensions. (Unit: m).

The traditional L-box test mold is mainly made of a steel plate, including a partition movable door and steel mesh. Considering the main measurement of the flow properties of concrete in this test program, the reinforcing mesh was removed beforehand, and a scale was attached to the inside and the surface of the mold to record the time of concrete flow in relation to each characteristic point under self-weight. The experimental apparatus and dimensions are shown in Figure 2.

Three parallel tests were conducted for each condition, and the results were averaged while ensuring that each test was valid. The results are shown below Table 7:

### 2.2. Verification of the Accuracy of Rheological Parameters

In this paper, on the basis of CFD Eulerian multiphase flow simulation technology, ANSYS FLUENT 2022R1 software (ANSYS, Pittsburgh, PA, USA) was used to simulate and analyze the concrete flow test. By comparing the results of the test above, it provided the rheological parameters that meet the requirements of field casting for the subsequent simulation.

A Eulerian multiphase flow model can achieve the simulation of particle flow process, however it is different from the traditional discrete model method. The Eulerian model regards the particulate matter as fluid, although the simulation method is still based on the concept of fluid, meaning that the Eulerian model cannot trace the particle trajectory like the discrete model. As a result, the concrete particles in the study of this paper were treated with fluidization, and there were some defects in expressing the flow and stacking characteristics of the concrete under different particle sizes, shapes, and gradations.

#### 2.2.1. Numerical Simulation of Slump Test

**Figure 3:**
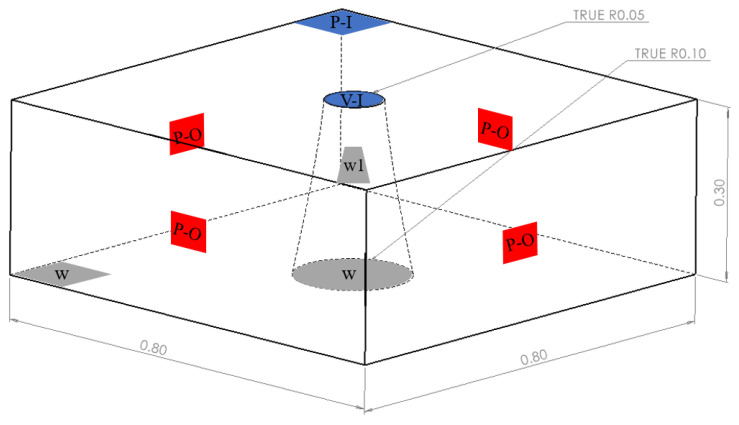
Slump test model dimensions and boundary settings.

In order to equally simulate the flow process of concrete in the actual test process, this paper used the SOLIDWORKS 2016 software (Dassault Systemes, MA, USA) to establish a conical table with a radius of the upper surface of 0.05 m, a radius of the lower bottom surface of 0.1 m, and a height of 0.3 m shown in the Figure 3 below as the initial filling area of the concrete. Based on the extended radius of the actual concrete, the calculation area of the rectangular model was set to a length of 0.8 m, a width of 0.8 m, and a height of 0.3 m, and was then divided into 187,562 cells with a grid quality greater than 0.5.

After a reasonable setting of the boundary conditions and determination of the discrimination method for calculating the stopping time, the slump state of concrete under the respective rheological parameters was simulated for the eight sets of slump experiments that had been carried out with a particle diameter of 5 mm. The total volume fraction of particles within the concrete was calculated to be roughly 0.4 according to the concrete mix ratio. The filling time of the concrete was selected to be 30 s, and the flow rate required for the injection of the mortar was calculated to be 0.023 m/s. The final results of the simulation are as follows Table 8:

The numerical solutions were found to be slightly larger than the test results by comparison, however they were all within a reasonable range. During the slump test, the filled mix was in direct contact with the cylinder wall, and since the cylinder wall was not an absolutely smooth wall, when the slump cylinder was lifted at a certain speed it would produce a certain perturbation to the initial state of the concrete. In contrast, in the state of numerical simulation, the lifting of the slump cylinder was just a process of changing the boundary conditions and there was no sign that the slump cylinder perturbs the concrete. This brought about the deviation of the numerical value from the test results.

Compared to the above deviations in boundary conditions, the computational characteristics of the Eulerian multiphase flow model were the most important source of discrepancies between numerical and experimental results. In the numerical simulation of concrete with large fluidity, the Eulerian method had some limitations in the basic assumption of treating solid particles as a proposed fluid, and the scheme was unable to simulate the real and complex distribution of particle sizes. Moreover, the Eulerian simulation ignored the size of the particles, and only considered the particle size factor in calculating the traction force between the particles and the mortar phase, which also lead to the deviation of the results to a large extent.

#### 2.2.2. Numerical Simulation of L-Box Tests

**Figure 4:**
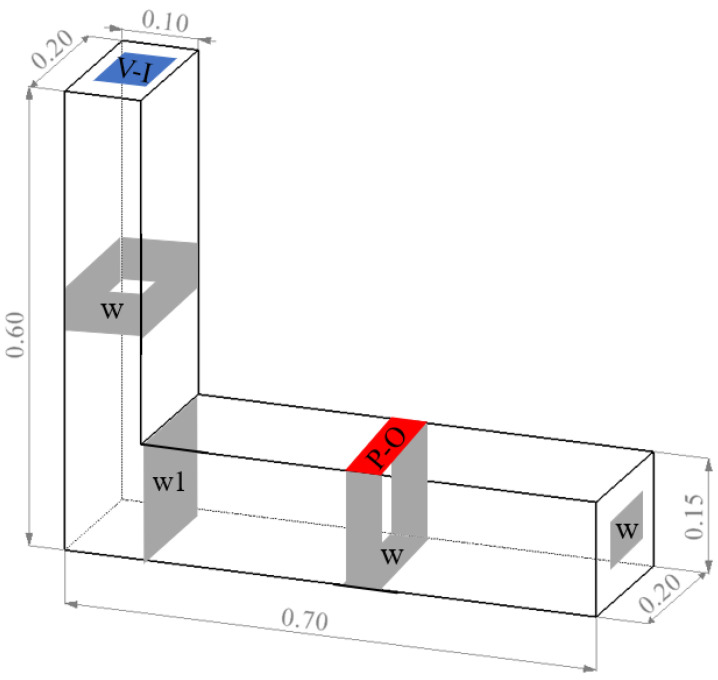
L-box test model dimensions and boundary settings.

Similarly to the slump simulation test, in order to equally simulate the flow process of concrete in the L-shaped box during the actual test, the author used the SOLIDWORKS 2016 software to establish an L-shaped space with a vertical size of 0.2 m × 0.1 m × 0.6 m on the left and 0.7 m × 0.2 m × 0.15 m on the right, as shown in the Figure 4 below. The left side was used as the initial filling area of concrete, and the right side was the flow area.

In order to ensure that the size of the divided grid cells had the least influence on the accuracy of the calculation results, it was divided into 240,000 cells and the quality of the grid was 1. The rest of the steps and the slump simulation were roughly similar. The simulation results are as follows Table 9:

The numerical solutions were generally lower than the experimental values and the flow of concrete was better in the numerical simulation process. The fact that the movable door of the spacer was not absolutely smooth caused the numerical simulation of the L-shaped flow test to ignore the disturbance of the initial state of the concrete caused by the lifting of the movable door of the spacer. Instead, it assumed that the movable door of the spacer disappeared directly, which brought about the deviation of the numerical value from the experimental results. In addition, the computational characteristics of the Eulerian model itself were the main source of error.

Comparing the test and simulation results of the slump extension and L-box, it can be seen that the difference between the data obtained from the two was insignificant and they were all within a reasonable range. Therefore, it can be concluded that the rheological parameters obtained through the rotational rheometer were accurate and can be used as the parameters of the subsequent simulation. Through comprehensive comparison, the rheological parameters of the M5, M7, and M8 cases with smaller errors can be selected as the simulation parameters as follows Table 10:

## 3. Simulation and Characterization of Circumferential Concrete Flow in Vaults

Much of the software that is currently on the market, including ANSYS FLUENT, often need to consume a huge amount of computational resources in the process of calculating fluid. For the actual lining structure of railroads and highway tunnels, the amount of the whole mold lining concrete is generally more than 80 m^3^, and full-size 3D pouring simulations are difficult to complete in a short period of time. In order to ensure the feasibility of the simulation, the simulation programs in this paper were simplified as follows:

(1) Considering the more realistic 2D simulation program, the vault area was divided into two regions: the vault ring direction and the vault longitudinal direction;
(2) In the process of the pouring simulation, enhancing the concrete flow performance by inserting vibrating rods and attaching vibrators was not considered;
(3) Only the effect of reinforcement perpendicular to the concrete flow direction in the lining on the concrete was considered.

### 3.1. Model Introduction and Boundary Condition Setting

**Figure 5:**
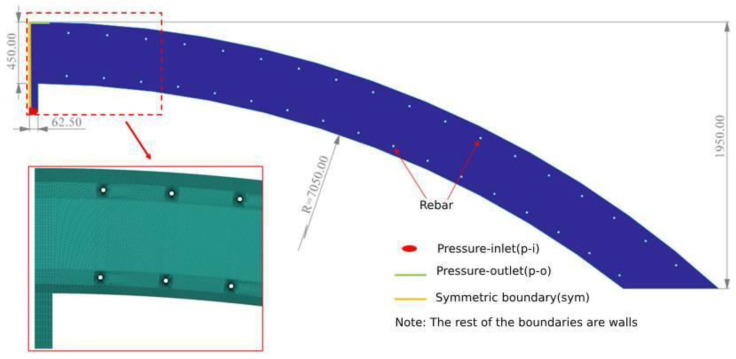
Vault ring casting model with mesh (R = 7.05 m).

In order to analyze the characteristics of the concrete circulation in the vault area during the lining casting, three casting models with different curvatures of 5.05 m, 6.05 m, and 7.05 m radius and 1.95 m height were made (Figure 5). The model was divided into 248,594 grid cells under the requirement of calculation accuracy. The left boundary was set as a symmetric boundary (sym) according to the actual pouring conditions, which simulated the circumferential flow of the concrete in the whole vault. The concrete in the vault was poured under pressure, so the filling hole below the model was set as pressure-inlet. The left boundary was set as a symmetric boundary (sym), and the pressure-outlet (1 atm) was set directly above the pouring hole. The rest of the boundaries were set as non-slipping walls.

### 3.2. Selection of Calculation Working Conditions

The radius size of the actual tunnel changes with the different factors such as the designed speed of the road surface and the number of required lanes. For this reason, the author not only analyzed the circumferential flow characteristics of concrete under different slumps and pumping pressures at the same radius, but also analyzed the flow characteristics of concrete under different radiuses. Three compliant concrete conditions as described in the previous section were selected taking into account the work of Zhang Minqing and other scholars. Considering the tunnel arch lining concrete pumping pressure control standards, pumping pressures of 10 kPa, 20 kPa, and 30 kPa were selected. In terms of the radiuses selected, 5.05 m, 6.05 m, and 7.05 m models were chosen for the simulation. The specific conditions are shown in the table below Table 11.

### 3.3. Concrete Flow Characteristics under Different Slumps

**Figure 6:**
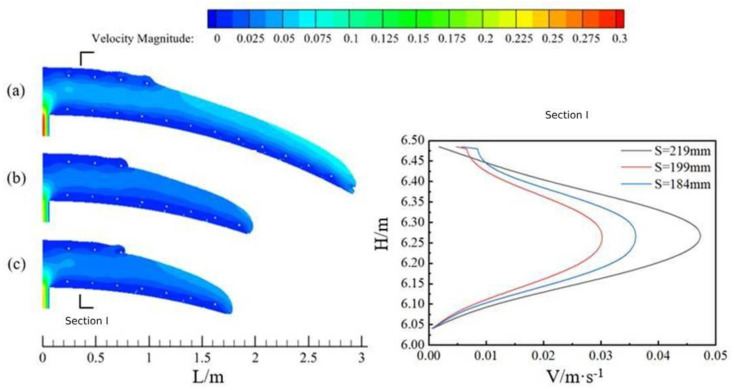
Characteristics of circumferential flow of concrete at three slumps and cross−sectional velocity profiles.

Figure 6 shows the flow state of concrete with three slumps (a for Case 1, slump 219 mm; b for Case 2, slump 199 mm; and c for Case 3, slump 184 mm) at the same time in the arch along the circumferential direction with the tunnel cross-section radius of 6.05 m and pumping pressure of 20 kPa.

From the point of view of the flow state of individual concrete, the concrete inside the model basically presented two major flow characteristics before and after. First of all, the concrete near the filling hole area was blocked by the upper and lower reinforcement, and its flow characteristics were similar to the flow characteristics of concrete in the pumping pipe studied by previous scholars [20]. The areas between the lowest reinforcement and the bottom plate and the uppermost reinforcement and the top plate were similar to the lubrication layer. The middle area was the core area and the whole cross-section (section I) showed the phenomenon of high flow rate in the middle and low flow rate on both sides. Secondly, the concrete at the frontmost end, because it was only blocked by the lowest layer of reinforcement and the friction effect of the base plate, showed the phenomenon of layered flow as a whole. The concrete on top, because of its greater speed, was gradually close to the base plate in the process of forward pouring. Its speed decreased, resulting in the realization of the forward transport of concrete. It can be seen that the filling process of concrete in the ring direction area of the vault was firstly filled along the ring direction, and when the flow reaches a certain slope, it started to be filled from the bottom to the top.

Comparing the flow state of concrete with three slumps at the same moment, it can be seen that under the same pumping pressure, the larger the slump of the concrete, the higher the flow rate of the overall cross-section and the farther the distance moved.

**Figure 7:**
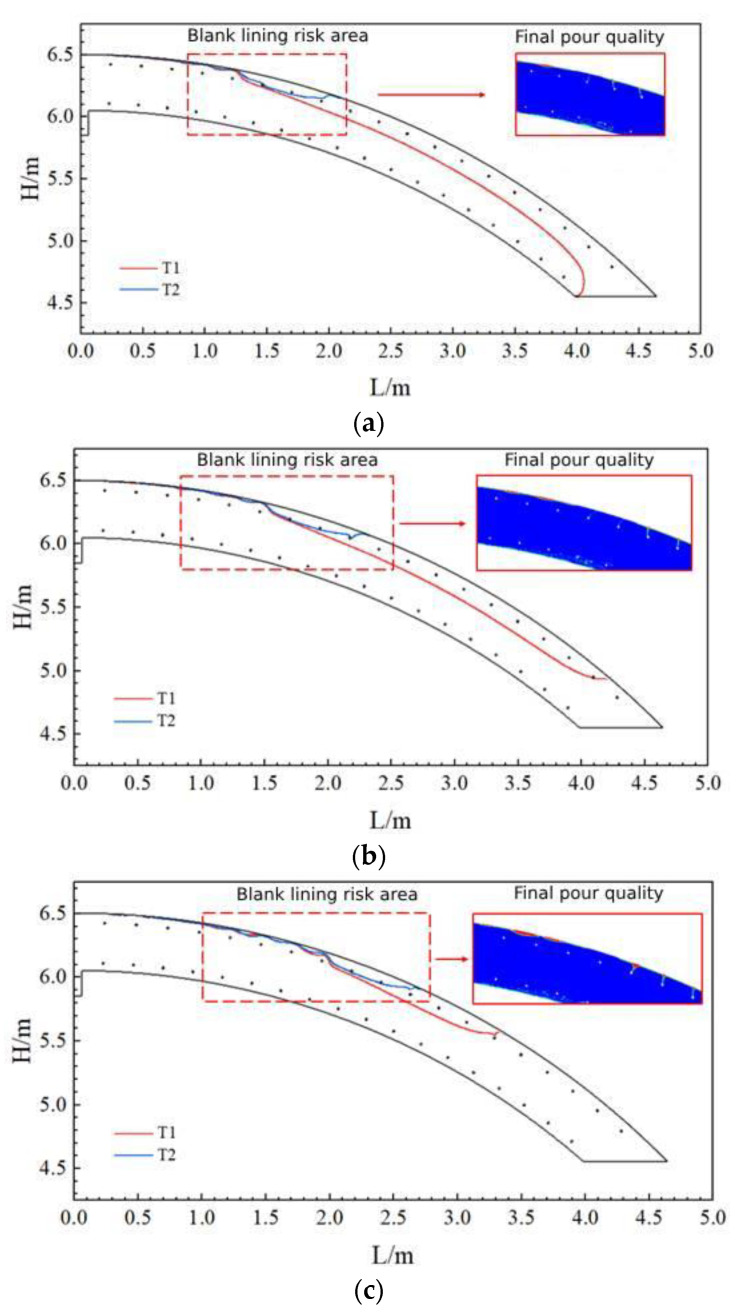
Movement distance and pouring results of the uppermost layer of concrete at three slumps: (**a**) Slump S = 219 mm; (**b**) Slump S = 199 mm; and (**c**) Slump S = 184 mm.

Figure 7 shows the maximum moving distance of the uppermost concrete at the beginning of pouring (T1) under the three slumps. When the concrete slump was 219 mm, the uppermost concrete moved the shortest distance (about 1.2 m); when the concrete slump was 184 mm, the uppermost concrete moved the farthest, reaching 1.9 m.

In addition, the figure also shows the area filled with concrete at the last moment (T2) and the quality of the filled area, respectively. According to the pouring quality of each model, when the concrete was poured uninterruptedly until the end, at the uppermost reinforcement, slight blank lining occurred in the vicinity of the maximum distance of concrete movement. The higher the slump, the higher the blank lining was located and the closer it was to the filling hole.

### 3.4. Characteristics of Concrete Circumferential Flow under Different Pumping Pressures

**Figure 8:**
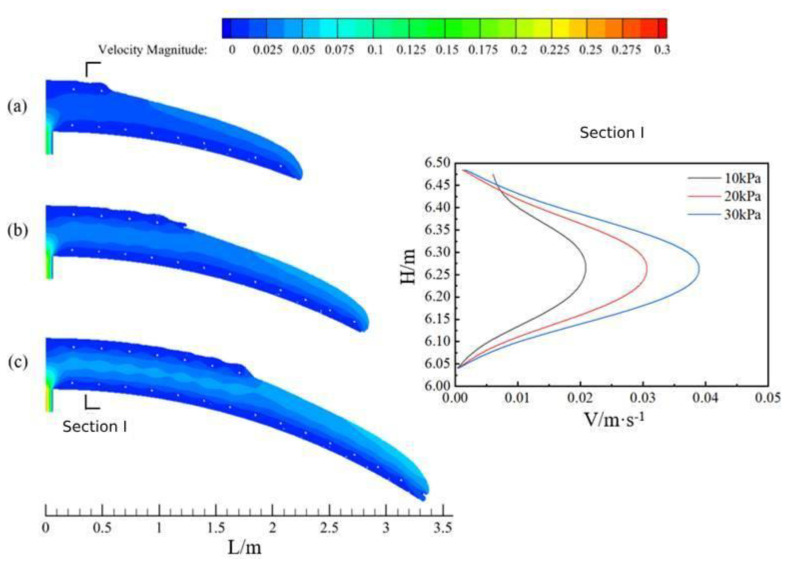
Characteristics of concrete circumferential flow and cross-sectional velocity profiles at three pumping pressures.

Figure 8 shows the flow of concrete along the ring direction in the arch at the same moment when the tunnel cross-section radius is 6.05 m and the concrete slump is 199 mm under three pumping pressures (a for Case 4, pumping pressure 10 kPa; b for Case 2, pumping pressure 20 kPa; and c for Case 5, pumping pressure 30 kPa). In terms of the flow characteristics of concrete, it was similar to the flow of concrete above.

Comparing the flow state of concrete at the same moment under the three pumping pressures, it can be seen that when the pumping pressure of concrete was increased in an equal sequence, the flow rate of the overall cross-section as well as the distance moved by the concrete also increased in an equal sequence.

**Figure 9:**
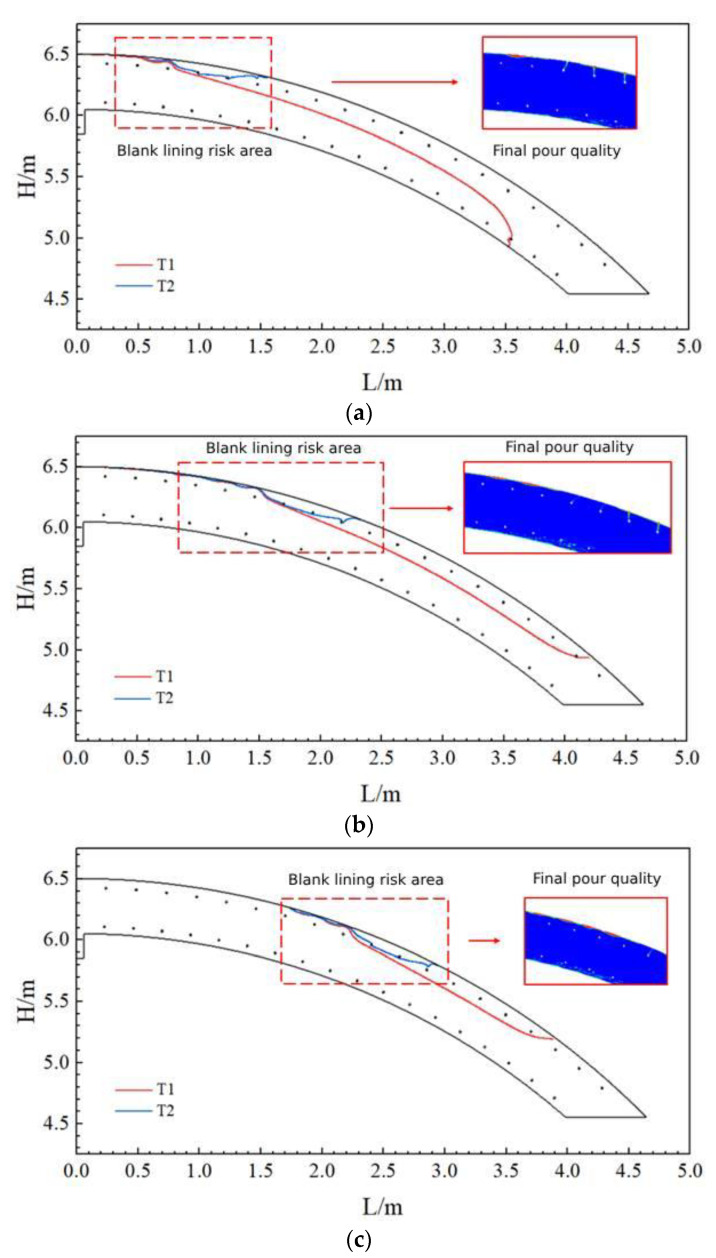
Movement distance and pouring results of the uppermost layer of concrete at three pumping pressures: (**a**) pumping pressure P = 10 kPa; (**b**) pumping pressure P = 20 kPa; and (**c**) pumping pressure P = 30 kPa.

Figure 9 shows the maximum movement distance of the uppermost layer of concrete at the beginning of pouring (T1) under the three pumping pressures of the aforementioned working conditions. When the concrete pumping pressure was 10 kPa, the uppermost layer of concrete moved the shortest distance (about 0.7 m); when the concrete pumping pressure was 30 kPa, the uppermost layer of concrete moved the farthest (up to 2.2 m). According to the figure showing the last moment of concrete (T2) filling and the quality of the filled area, when the concrete was poured uninterruptedly until the end, slight blank lining still occurred in the vicinity of the maximum distance of concrete flow at the uppermost reinforcement. The lower the pumping pressure, the higher the location of the blank lining area, and the closer it was to the filling hole.

### 3.5. Concrete Circumferential Flow Characteristics under Different Lining Radius

**Figure 10:**
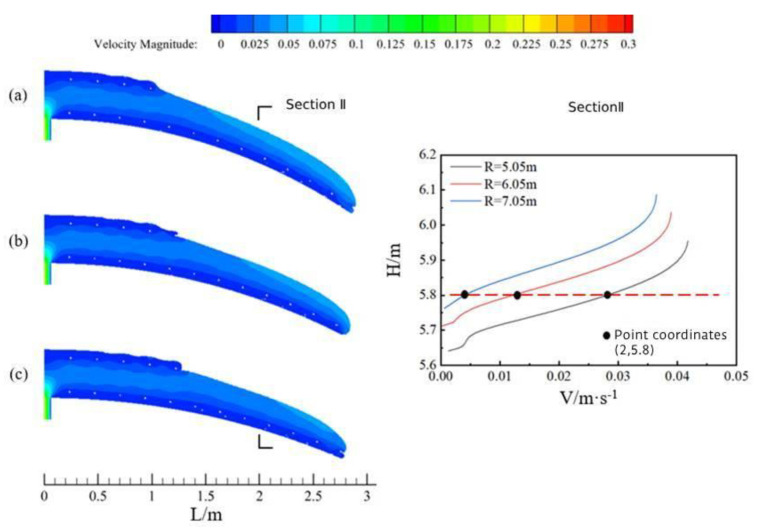
Characteristics of circumferential concrete flow and cross-section velocity profiles at three tunnel cross-section radiuses.

Figure 10 shows the concrete flow state along the ring direction in the arch at the same moment under three tunnel cross-section radius R (a for Case 6, radius 5.05 m; b for Case 2, radius 6.05 m; and c for Case 7, radius 7.05 m) when the concrete slump is 199 mm and the pumping pressure is 20 kPa. As far as the flow characteristics of the concrete were concerned, they were also similar to those of the concrete above.

Comparison of the concrete flow at the same moment in time for the three tunnel cross-section ratios showed that as the radius of the tunnel cross-section increased, a slight decrease in the flow velocity of the overall cross-section as well as in the distance that the concrete moved horizontally also occurred.

**Figure 11:**
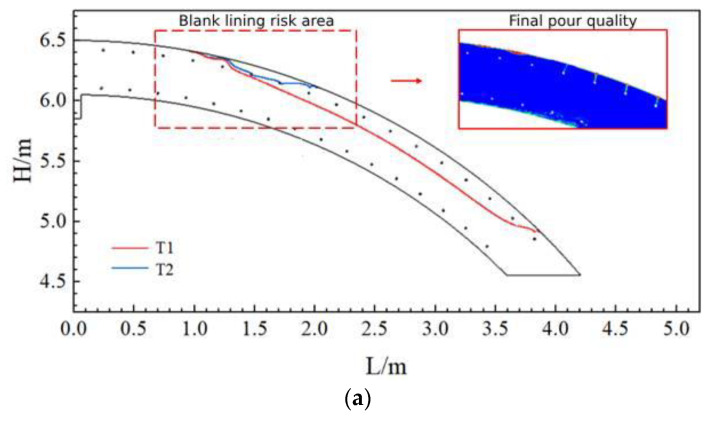

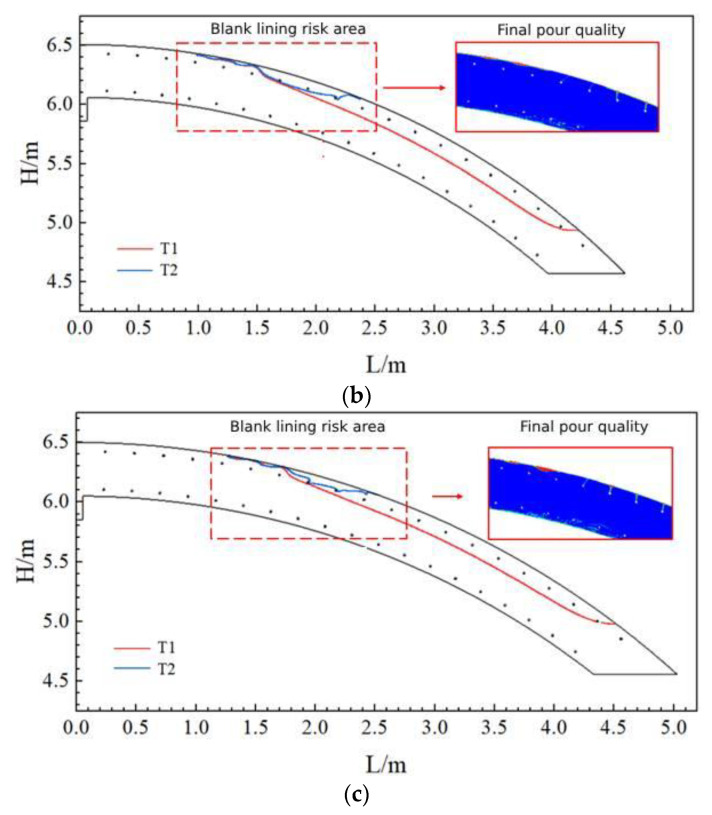
Movement distance and casting effect of the uppermost concrete layer for three tunnel cross-section radiuses: (**a**) section radius R = 5.05 m; and (**b**) section radius R = 6.05 m; and (**c**) section radius R = 7.05 m.

Figure 11 shows the maximum movement distance of the uppermost layer of concrete at the beginning of pouring (T1) for the three tunnel cross-section radius. When the tunnel cross-section radius was 5.05 m, the uppermost concrete moved the shortest distance (about 1.3 m); when the tunnel cross-section radius was 7.05 m, the uppermost concrete moved the furthest, reaching 1.7 m. Based on the area filled at the last moment of concrete (T2) and the quality of the filled area shown in the figure, when the concrete was poured uninterruptedly until the end of the pour, slight blank lining still occurred in the vicinity of the maximum distance of concrete flow at the uppermost reinforcement. The smaller the radius, the higher the blank lining was located, and the closer it was to the filling hole.

## 4. Simulation and Characterization of Longitudinal Concrete Flow in Vaults

### 4.1. Model Introduction and Boundary Condition Setting

**Figure 12:**
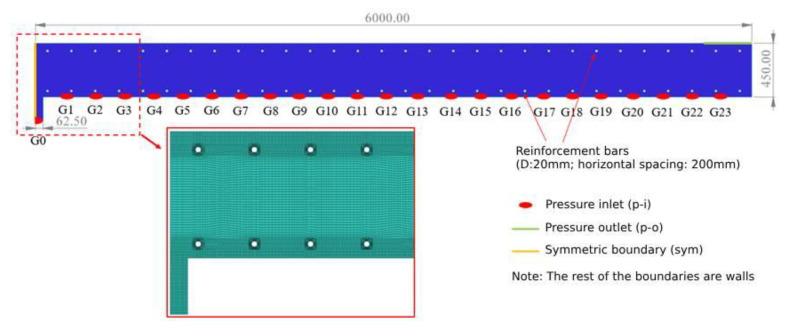
Longitudinal casting model and mesh for vaults.

In order to analyze the flow characteristics of concrete along the longitudinal direction of the vault, a casting model with a length of 6 m and a thickness of 0.45 m was made (Figure 12), and the model was set up with a 0.125 m-diameter pouring hole (G0–G23) every 0.25 m. The model was divided into 189,826 grid cells under the requirement of calculation accuracy. According to the actual casting conditions and working condition requirements, each pouring hole was set as a pressure-inlet or a non-slipping wall. The left boundary was set as a symmetric boundary (sym), the upper right boundary was set as a pressure-outlet (1 atm), and the rest of the boundaries were set as non-slipping walls.

### 4.2. Selection of Calculation Working Conditions

When analyzing the longitudinal flow characteristics of concrete in the vault, the simulation scheme only set the G0 filling hole as the pressure inlet, with the rest were being classified as wall surfaces. The author selected the three different slumps of concrete described in the previous section and conformed to the requirements of tunnel lining design and pumping specifications. The flow characteristics of concrete under three pumping pressures of 10 kPa, 20 kPa, and 30 kPa were analyzed, and the specific working conditions are shown in Table 12.

### 4.3. Longitudinal Flow Characteristics of Concrete under Different Slumps

**Figure 13:**
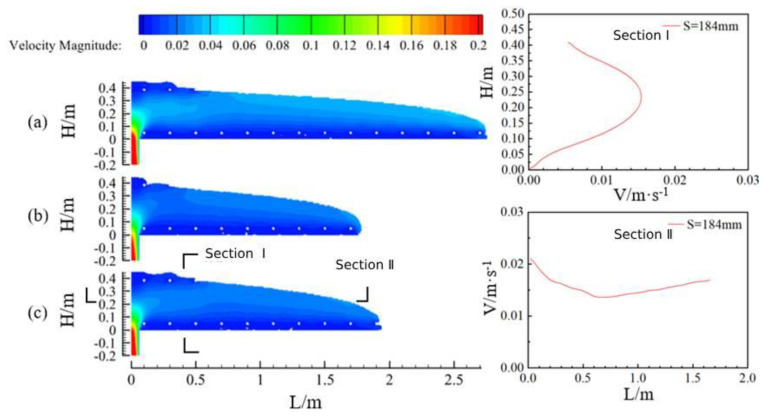
Longitudinal flow characteristics and cross-sectional velocity profiles of concrete at three slumps.

Figure 13 shows the flow state along the longitudinal direction of the vault at the same moment when the pumping pressure is 20 kPa for concrete with three slumps (a for Case 1, slump 219 mm; b for Case 2, slump 199 mm; and c for Case 3, slump 184 mm). Similarly, in the region between the filling hole and where concrete fills the uppermost reinforcement, the flow characteristics of the concrete were similar to those of the concrete in the pumping pipe, which showed a high velocity in the core and a low velocity in the lubrication zone (Section I). In the foremost end of the concrete casting, the concrete flowed in layers. The concrete near the pouring hole had a higher flow rate under the action of larger pressure, the concrete in the foremost free interface area was subjected to less resistance and had a higher flow rate, and the concrete in the middle was squeezed by the concrete in the front end with a lower flow rate. This means that in the horizontal direction, with the increase of the distance, the flow rate of the concrete showed the phenomenon of decreasing first and then increasing (Section II).

**Figure 14:**
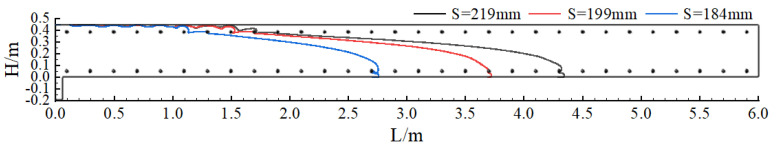
Maximum flow distance of concrete at three slumps.

According to scholars’ research and the simulation results in the previous section, it can be seen that when the flow rate of concrete is lower than 0.002 m/s, it can be determined that the concrete flow has stopped, and it is not possible to continue to fill the concrete into the model under the same pressure. Figure 14 shows the maximum movement distance of concrete under three kinds of slump. It can be seen that under the same pumping pressure conditions, the larger the slump of the concrete, the farther the pumping distance. When the slump was 219 mm, the maximum pumping distance reached 4.35 m, meaning that the pumping effect was the best. When the slump was 184 mm, the maximum pumping distance was 2.80 m, meaning that the pumping effect was poor.

### 4.4. Longitudinal Flow Characteristics of Concrete under Different Pumping Pressures

**Figure 15:**
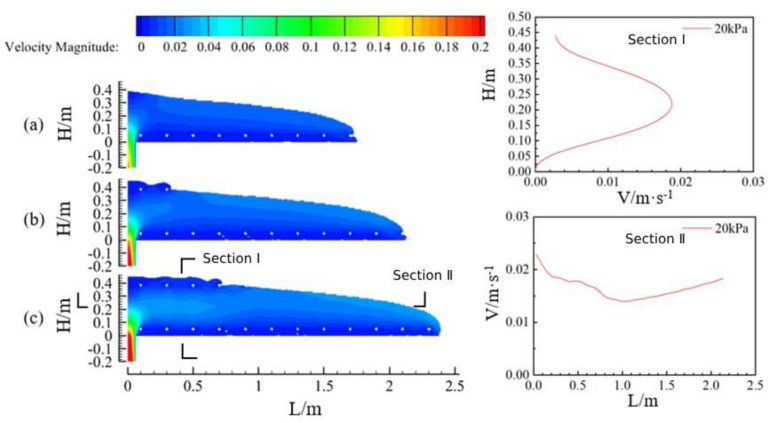
Characteristics of longitudinal concrete flow and cross-section velocity profiles under three pumping pressures.

Figure 15 shows the flow of concrete along the longitudinal direction of the vault at the same moment for three pumping pressures (a for Case 4, pumping pressure 10 kPa; b for Case 2, pumping pressure 20 kPa; and c for Case 5, pumping pressure 30 kPa) when the concrete slump is 199 mm. In terms of the flow characteristics of concrete, it is similar to the flow of concrete above. Comparing the flow state of concrete at the same moment under three pumping pressures, it can be seen that with the increase of pumping pressure, the flow rate of concrete also increased.

**Figure 16:**
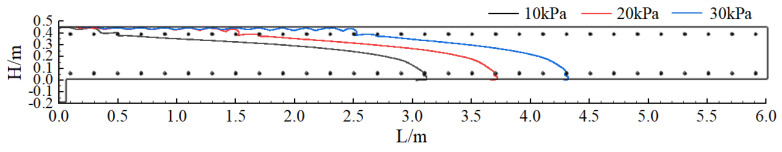
Maximum flow distance of concrete at three pumping pressures.

Figure 16 shows the maximum moving distance of concrete under three pumping pressures. For the same kind of slump concrete, the higher the pumping pressure, the farther the concrete was pumped. When the pumping pressure was 30 kPa, the maximum pumping distance reached 4.3 m, meaning that at this time the pumping effect was the best. When the pumping pressure was 10 kPa, the maximum pumping distance was 3.1 m, meaning that at this time the pumping effect was poor.

## 5. Conclusions

This paper focused on the simulation of the circumferential and longitudinal flow of concrete under pressurized feeding conditions during tunnel vault casting. The main conclusions are as follows:

1. The circumferential and longitudinal flow of concrete in the vault shows two major flow characteristics before and after. The flow characteristics near the filling hole area are similar to the flow characteristics of the concrete in the pumping pipe. The area between the lowest reinforcement and the bottom plate and the uppermost reinforcement and the top plate is the lubrication layer, and has a low flow rate. The middle is the core area, and has a high flow rate. The concrete at the front end presents a layered flow phenomenon as a whole, and the upper layer of the concrete has a higher speed. It gradually gets closer to the bottom plate and the speed is reduced during the forward pouring process and achieves the forward transport of concrete.
2. In the process of vault casting, the larger the concrete slump, the larger the pumping pressure. The smaller the radius of the tunnel, the higher the flow rate of the overall cross-section at the same moment, and the farther the concrete movement. Therefore, in the actual project, according to the tunnel radius, concrete slump, pumping pressure, and other construction conditions can be the reasonable design of longitudinal filling hole spacing.
3. During the casting process of the arch, the region from the arch to the arch shoulder was prone to the risk of blank lining. The higher the slump of the concrete, the lower the pumping pressure. Also, the smaller the radius of the tunnel cross-section, the higher the blank lining risk zone will be, and the closer it will be to the filling hole. Therefore, in order to avoid the phenomenon of blank lining during the casting of the secondary lining of the tunnel, optimization of the position of the uppermost pouring window of the lining trolley (by appropriately elevating the window position) must occur.

## Figures and Tables

**Table 1:** Main performance indicators of cement.

Density (g/cm^3^)	Specific Surface Area (m^2^/kg)	Water Consumption for Standard Consistency (%)	Condensation Time (min)		Stability	28 d Compressive Strength (Mpa)
			Initial Setting	Final Setting		
3.15	360	28	175	235	Eligible	49

**Table 2:** Main performance index of medium sand.

Modulus of Fineness	Mud Content (%)	Apparent Density (kg/m^3^)	Stacking Density (kg/m^3^)	Dense Voids (%)
2.73	1.61	2602.6	1670.8	35.8

**Table 3:** Main properties of fly ash.

Density (g/cm^3^)	Specific Surface Area (m^2^/kg)	Water Requirement Ratio (%)	45 μm Sieve Residue (%)	Stability	Loss on Burnout (%)
2.15	450	103	18	Eligible	6.73

**Table 4:** Concrete mix ratio under each working condition.

Grouping Number	Cement	Admixture	Fine Aggregate	Coarse Aggregate 1	Coarse Aggregate 2	Additives	Water	Water-Cement Ratio	Sand Ratio	Water Reducing Agent Dosage
M1	291	125	749	323	749	4.16	178	0.43	0.41	1%
M2	299	128	749	323	749	4.16	167	0.39	0.41	1%
M3	294	127	749	323	749	4.16	173	0.41	0.41	1%
M4	287	123	749	323	749	4.16	184	0.45	0.41	1%
M5	283	122	749	323	749	4.16	189	0.47	0.41	1%
M6	291	125	674	345	802	4.16	178	0.43	0.37	1%
M7	291	125	710	334	777	4.16	178	0.43	0.39	1%
M8	291	125	783	312	726	4.16	178	0.43	0.43	1%

**Table 5:** Test results of rheological parameters of concrete in each working condition.

Grouping Number	$\mathrm{Yield}\;\mathrm{Stress}\;\tau_{0}$ (Pa)	$\mathrm{Plastic}\;\mathrm{Viscosity}\;\mu_{p}$ (Pa·s)
M1	140	136
M2	687	139
M3	447	142
M4	188	127
M5	126	107
M6	926	438
M7	544	176
M8	312	225

**Table 6:** Test results of slump extension of concrete for each working condition.

Grouping Number	Mean Value of Slump S (mm)	$\mathrm{Mean}\;\mathrm{Value}\;\mathrm{of}\;\mathrm{Extension}\;D_{f}$ (mm)
M1	201	503
M2	165	459
M3	181	477
M4	208	515
M5	219	529
M6	173	470
M7	184	491
M8	199	517

**Table 7:** Concrete L-box flow test results under different working conditions.

Grouping Number	T_200_ (s)	T_300_ (s)	T_400_ (s)	T_500_ (s)	T_600_ (s)	T_700_ (s)
M1	2.5	3.9	5.3	6.0	8.2	11.5
M2	3.1	5.4	7.3	10.9	19.1	32.2
M3	2.5	3.4	4.7	7.5	12.4	23.7
M4	2.2	3.1	4.3	5.8	8.1	11.4
M5	1.8	2.7	3.8	5.0	6.1	7.3
M6	3.4	6.3	11.5	21.0	35.8	-
M7	3.3	5.1	8.7	13.9	21.5	30.4
M8	2.6	4.5	6.8	10.6	16.1	22.3

**Table 8:** Slump extension test results and numerical results.

Test Condition	Slump (mm)			Extension (mm)		
	Test Value	Numerical Solution	Error (%)	Test Value	Numerical Solution	Error (%)
M1	201	218	8.5	503	523	4.0
M2	165	190	15.2	459	479	4.4
M3	181	202	11.6	477	490	2.7
M4	208	223	7.2	515	522	1.4
M5	219	229	4.6	529	524	0.9
M6	173	194	12.1	470	478	1.7
M7	184	199	8.2	491	521	6.1
M8	199	208	4.5	517	537	3.9

**Table 9:** L-box test results and numerical results.

Condition	Projects	T_200_ (s)	T_300_ (s)	T_400_ (s)	T_500_ (s)	T_600_ (s)	T_700_ (s)
M1	Test Value	2.5	3.9	5.3	6.0	8.2	11.5
	Numerical Solution	0.6	1.3	2.4	3.5	5.3	9.4
M2	Test Value	3.1	5.4	7.3	10.9	19.1	32.2
	Numerical Solution	0.9	2.1	3.8	7.7	15.4	26.8
M3	Test Value	2.5	3.4	4.7	7.5	12.4	23.7
	Numerical Solution	0.8	1.8	3.1	5.4	10.4	19.0
M4	Test Value	2.2	3.1	4.3	5.8	8.1	11.4
	Numerical Solution	0.6	1.3	2.3	3.5	5.6	10.3
M5	Test Value	1.8	2.7	3.8	5.0	6.1	7.3
	Numerical Solution	0.5	1.1	1.9	2.9	4.3	7.6
M6	Test Value	3.4	6.3	11.5	21.0	35.8	-
	Numerical Solution	1.6	4.2	8.3	15.2	26.1	41.1
M7	Test Value	3.3	5.1	8.7	13.9	21.5	30.4
	Numerical Solution	0.9	2.1	3.7	7.1	13.8	24.2
M8	Test Value	2.6	4.5	6.8	10.6	16.1	22.3
	Numerical Solution	0.9	2.0	3.3	5.6	10.3	18.0

**Table 10:** Table of parameter values for numerical simulation of vault concrete.

Grouping Number	Yield Stress (Pa)	$\mathrm{Plastic}\;\mathrm{Viscosity}\;\mu_{p}$ (Pa·s)	Slump (mm)	Extension (mm)
M5	126	107	219	529
M7	544	176	184	491
M8	312	225	199	517

**Table 11:** Lining vault ring direction simulation calculation working conditions.

Cases	$\mathrm{Yield}\;\mathrm{Stress}\;\tau_{0}$ (Pa)	$\mathrm{Plastic}\;\mathrm{Viscosity}\;\mu_{p}$ (Pa·s)	Model Radius (m)	Pumping Pressure (kpa)
Case1	126	107	6.05	20
Case2	312	225	6.05	20
Case3	544	176	6.05	20
Case4	312	225	6.05	10
Case5	312	225	6.05	30
Case6	312	225	5.05	20
Case7	312	225	7.05	20

**Table 12:** Longitudinal simulation of lining vault calculation conditions.

Cases	$\mathrm{Yield}\;\mathrm{Stress}\;\tau_{0}$ (Pa)	$\mathrm{Plastic}\;\mathrm{Viscosity}\;\mu_{p}$	Pumping Pressure (kpa)
Case1	126	107	20
Case2	312	225	20
Case3	544	176	20
Case4	312	225	10
Case5	312	225	30

## Data Availability

All data are available from the authors.

## References

[1] Afshani A., Kawakami K., Konishi S., Akagi H. (2019). Study of Infrared Thermal Application for Detecting Defects within Tunnel Lining. Tunn. Undergr. Space Technol..

[2] Jiang Y., Wang L., Zhang B., Dai X., Ye J., Sun B., Liu N., Wang Z., Zhao Y. (2023). Tunnel Lining Detection and Retrofitting. Autom. Constr..

[3] Rosso M.M., Marasco G., Aiello S., Aloisio A., Chiaia B., Marano G.C. (2023). Convolutional Networks and Transformers for Intelligent Road Tunnel Investigations. Comput. Struct..

[4] Protopapadakis E., Voulodimos A., Doulamis A., Doulamis N., Stathaki T. (2019). Automatic Crack Detection for Tunnel Inspection Using Deep Learning and Heuristic Image Post-Processing. Appl. Intell..

[5] Yao Q., Qi S., Wu F., Yang X., Li H. (2020). Abrasion-Resistant and Temperature Control of Lining Concrete for Large-Sized Spillway Tunnels. Appl. Sci..

[6] Oreste P., Spagnoli G., Luna Ramos C.A., Hedayat A. (2019). Assessment of the Safety Factor Evolution of the Shotcrete Lining for Different Curing Ages. Geotech. Geol. Eng..

[7] Hao S., Fei R. (2022). Optimisation Study on Crack Resistance of Tunnel Lining Concrete Under High Ground Temperature Environment. Geotech. Geol. Eng..

[8] Zhang Z., Chen B., Li H., Zhang H. (2022). The Performance of Mechanical Characteristics and Failure Mode for Tunnel Concrete Lining Structure in Water-Rich Layer. Tunn. Undergr. Space Technol..

[9] Han W., Jiang Y., Wang G., Liu C., Koga D., Luan H. (2023). Review of Health Inspection and Reinforcement Design for Typical Tunnel Quality Defects of Voids and Insufficient Lining Thickness. Tunn. Undergr. Space Technol..

[10] Iskhakov T., Timothy J.J., Plückelmann S., Breitenbücher R., Meschke G. (2023). Compressible Cementitious Composite Materials: Multiscale Modeling and Experimental Investigation. Cem. Concr. Compos..

[11] Moradi P., Asadi M.J., Ebrahimzadeh N., Yarahmadi B. (2021). Ilam Tunnels Inspection, Maintenance, and Rehabilitation: A Case Study. Tunn. Undergr. Space Technol..

[12] Wu Y., Shao S., Fu H., Liu D., Liu C., Ma Z. (2019). Analysis of Tunnel Lining Vault Void and Methods of Active Monitoring and Prevention. China Saf. Sci. J..

[13] Krenzer K., Mechtcherine V., Palzer U. (2019). Simulating Mixing Processes of Fresh Concrete Using the Discrete Element Method (DEM) under Consideration of Water Addition and Changes in Moisture Distribution. Cem. Concr. Res..

[14] Kolařík F., Patzák B., Zeman J. (2018). Computational Homogenization of Fresh Concrete Flow around Reinforcing Bars. Comput. Struct..

[15] Haustein M.A., Eslami Pirharati M., Fataei S., Ivanov D., Jara Heredia D., Kijanski N., Lowke D., Mechtcherine V., Rostan D., Schäfer T. (2022). Benchmark Simulations of Dense Suspensions Flow Using Computational Fluid Dynamics. Front. Mater..

[16] Mechtcherine V., Bos F.P., Perrot A., Da Silva W.R.L., Nerella V.N., Fataei S., Wolfs R.J.M., Sonebi M., Roussel N. (2020). Extrusion-Based Additive Manufacturing with Cement-Based Materials—Production Steps, Processes, and Their Underlying Physics: A Review. Cem. Concr. Res..

[17] Caliendo C., Russo I. (2022). A 3D Computational Fluid Dynamics Model for Assessing the Concrete Spalling of a Tunnel Lining in the Event of a Fire. Comput. Geotech..

[18] Chen J., Martin P., Xu Z., Manzano H., Dolado J.S., Ye G. (2021). A Dissolution Model of Alite Coupling Surface Topography and Ions Transport under Different Hydrodynamics Conditions at Microscale. Cem. Concr. Res..

[19] Sassi R., Jelidi A., Montassar S. (2023). Numerical Simulation of Fresh Concrete Flow in the L-Box Test Using Computational Fluid Dynamics. Mag. Concr. Res..

[20] Gu Q. (2022). Investigation on Flow Characteristics of Wet Concrete Conveyed by Compressed Gas and Anti-Blocking of Pipeline Vibration. Master’s Thesis.

